# Estimating the undetected burden and the likelihood of strain persistence of drug-resistant *Neisseria gonorrhoeae*

**DOI:** 10.1093/aje/kwae455

**Published:** 2024-12-13

**Authors:** Kirstin I Oliveira Roster, Minttu M Rönn, Heather Elder, Thomas L Gift, Kathleen A Roosevelt, Joshua A Salomon, Katherine K Hsu, Yonatan H Grad

**Affiliations:** Department of Immunology and Infectious Diseases, Harvard T.H. Chan School of Public Health, Boston, MA 02115, United States; Division of STD Prevention and HIV Surveillance, Massachusetts Department of Public Health, Boston, MA 02130, United States; Department of Global Health and Population, Harvard T. H. Chan School of Public Health, Boston, MA 02115, United States; Division of STD Prevention and HIV Surveillance, Massachusetts Department of Public Health, Boston, MA 02130, United States; Division of STD Prevention, Centers for Disease Control and Prevention, Atlanta, GA 30333, United States; Division of STD Prevention and HIV Surveillance, Massachusetts Department of Public Health, Boston, MA 02130, United States; Department of Health Policy, Stanford University School of Medicine, Stanford, CA 94305, United States; Division of STD Prevention and HIV Surveillance, Massachusetts Department of Public Health, Boston, MA 02130, United States; Section of Pediatric Infectious Diseases, Boston Medical Center 02118, Boston, MA, United States; Department of Immunology and Infectious Diseases, Harvard T.H. Chan School of Public Health, Boston, MA 02115, United States

**Keywords:** *Neisseria gonorrhoeae*, gonorrhea, antimicrobial resistance, pathogen surveillance, mathematical modeling

## Abstract

*Neisseria gonorrhoeae* has developed resistance to all antibiotics recommended for treatment, and reports of reduced susceptibility to ceftriaxone, the last-line treatment, are increasing. Because many asymptomatic infections remain undiagnosed and most diagnosed infections do not undergo antibiotic susceptibility testing, surveillance systems may underestimate resistant infections. In this modeling study, we simulated the spread of a new strain of ceftriaxone-nonsusceptible *N. gonorrhoeae* in a population comprising men who have sex with men as well as heterosexual men and women. We compared scenarios with varying strain characteristics and surveillance capacity. For each scenario, we estimated 1) the number of undetected infections of the novel strain and 2) the likelihood of strain persistence in the absence of newly reported cases. Upon detection of 1 nonsusceptible isolate, the undetected burden was an estimated 5.4 infections with substantial uncertainty (95% uncertainty interval, 0-18 infections). Without additional reports of nonsusceptible infections over the subsequent 180 days, the estimate declined to 2.5 infections (95% uncertainty interval, 0-10). The likelihood of ongoing transmission also declined from 66% (95% uncertainty interval, 26-86) at first detection to 2% (95% uncertainty interval, 0-10) after 180 days. To extend the useful lifespan of last-line antibiotics, our model estimated the infection landscapes that could underlie data from surveillance systems.

## Introduction

The urgent public health threat of antimicrobial resistance (AMR) demands new strategies to slow and control its spread. An estimated 1.27 million people died of resistant bacterial infections in 2019 globally.^1^ Especially concerning are increasing rates of resistance to last-line antibiotics^2^ and resistance in pathogens with high incidence, such as *Mycobacterium tuberculosis, Salmonella,* and *Neisseria gonorrhoeae.*^3^  *N. gonorrhoeae* causes an estimated 82 million infections annually^4^ and has developed resistance to all antibiotics recommended for treatment.^5^ Although novel therapeutics are in development,^6^^,^^7^ reports of resistance to ceftriaxone, the last approved treatment, are increasing,^8^^-^^12^ including in the United States.^13^

Two cases of an *N. gonorrhoeae* strain with reduced susceptibility to ceftriaxone and resistance or reduced susceptibility to all other approved anti-gonococcal antibiotics were identified in Massachusetts in 2022.^14^ After serendipitous discovery of the first case through urine culture, the Massachusetts Department of Public Health (MDPH) collaborated with the originating microbiology laboratory and the US Centers for Disease Control and Prevention (CDC) to perform retrospective molecular testing of remnant specimens from nucleic acid amplification tests conducted between January and September 2022; a total of 54 remnant specimens were tested, leading to the discovery of a second case from the same community health center from which the first nonsusceptible case was identified. The MDPH also issued a clinical alert (January 2023) to encourage increased gonococcal culture for symptomatic patients across Massachusetts.^14^^,^^15^ The expanded culture surveillance has yet to identify any additional ceftriaxone nonsusceptible cases.

However, many asymptomatic infections are undiagnosed,^16^^-^^18^ and the vast majority of diagnosed gonococcal infections do not undergo antibiotic susceptibility testing.^19^ Even after the clinical alert, culture isolates associated with only 5.5% of all nucleic acid amplification test–positive gonococcal cases in Massachusetts were submitted to the MDPH (*n* = 540 specimens from 9859 cases in the first year after the clinical alert release).^20^ Consequently, surveillance systems may underestimate the number of ceftriaxone-nonsusceptible cases. There is an urgent need for better interpretation and use of surveillance data to estimate the unseen prevalence of resistance.

In this modeling study, we sought to understand how surveillance signals relate to the true infection burden, including how much time must pass without detecting new cases of the novel strain before being confident there is no ongoing undetected transmission. To do so, we developed a mathematical model of gonorrhea transmission in Massachusetts among men who have sex with men (MSM), men who have sex with men and women, men who have sex with women, and women who have sex with men. We further split each group into 2 sexual activity classes with either high or low partner-change rates and calibrated mixing parameters to MDPH surveillance data. The model accounted for the natural history of gonococcal infection, care seeking behavior, and surveillance of AMR. We simulated the introduction of a ceftriaxone-nonsusceptible case and measured the number of unreported infections and the likelihood of unobserved transmission for different surveillance scenarios, including the reported scenario in Massachusetts in which no cases have been found since detection of 2 ceftriaxone-nonsusceptible infections in 2022. As sensitivity analyses, we examined scenarios in which we varied treatment failure rates of the nonsusceptible strain and rates of asymptomatic screening and antibiotic susceptibility testing.

This study provides a roadmap for situations in which increased resistance to the last-line antibiotic is observed globally and surveillance systems are tasked with tracking early introductions and emergence of resistance locally. Improved interpretation of gonorrhea surveillance data can help guide surveillance systems of AMR more broadly. Monitoring population prevalence of antibiotic resistance is fundamental to ensure treatment guidelines are compatible with the resistance profiles of circulating strains, to respond quickly to rising levels of resistance, and to extend the clinically useful lifespan of last-line antibiotics.^3^^,^^19^^,^^21^^-^^23^

## Methods

### Transmission model

We developed a stochastic susceptible-infected-susceptible compartmental model of gonorrhea transmission in Massachusetts (Figure S1, Appendix S1). We accounted for symptomatic and asymptomatic infections with either a ceftriaxone-susceptible or nonsusceptible strain of gonorrhea. In the context of this model, we defined strains in terms of their antibiotic susceptibility patterns, distinguishing specifically between those susceptible and nonsusceptible to treatment with ceftriaxone 500 mg. We assumed there were no infections with multiple strains of *N. gonorrhoeae*. The population was stratified into 8 subgroups based on gender identity, gender identity of sex partners, and 2 sexual activity groups with either high or low partner-change rates.

Gonococcal infections were diagnosed in response to symptomatic care seeking or asymptomatic screening, which resulted in treatment with the recommended regimen of ceftriaxone 500 mg. Resistance was monitored through antibiotic susceptibility testing of a subset of gonorrhea-positive isolates (from both symptomatic and asymptomatic infections), whereby not only the infection but also the strain causing the infection was identified by the surveillance system. Rates of asymptomatic screening (by gender, gender of sex partners, and sexual activity group) and antibiotic susceptibility testing (by gender) were among the parameters fitted to surveillance data in the main analysis (Table S1). In sensitivity analyses, we evaluated 1) rates of antibiotic susceptibility testing ranging from 1% to 40% of detected cases and 2) screening frequency with up to 2-fold increases (preserving the relative screening intensity between groups).

We assumed treatment of ceftriaxone-nonsusceptible infections results in treatment failure in 80% of infections. In sensitivity analyses, we considered treatment failure rates of 30% to 90%. This partial treatment failure is concordant with the 2 cases detected in Massachusetts, because these were successfully treated with ceftriaxone, the first case with 500 mg and the second case with 1 g plus 1.2 g of azithromycin.^14^ We assumed identification of treatment failure (either through persistent symptoms or test of cure of asymptomatic infections) would trigger antibiotic susceptibility testing, leading to the detection of the nonsusceptible infection, and treatment with an alternative curative antibiotic. Asymptomatic cases that were not followed up with a test of cure remained infectious until natural recovery. Treatment of infections with the susceptible strain was always successful.

### Model calibration

The model was calibrated to gonorrhea diagnosis rates by gender and prevalence among MSM, men who have sex with women, and women who have sex with men, using an approximate Bayesian computation rejection sampling approach with 1 million iterations (see Table S2 for starting conditions and Table S3 for calibration targets). Prior distributions of model parameters were defined using surveillance data from MDPH, where available, and values from the literature otherwise (see Table S1 for prior and posterior parameter distributions of fitted parameters and Table S2 for fixed parameters). Calibration was performed for the ceftriaxone-susceptible strain only.

### Introduction of nonsusceptible strain

After calibration of the ceftriaxone-susceptible strain dynamics, we introduced a single index case of the nonsusceptible strain into 1 of the 8 population subgroups and ran 500 simulation iterations for each subgroup. Sensitivity analyses were performed on the calibrated model. We iteratively filtered simulations that were consistent with surveillance scenarios in which detection of the first case was followed by increasing periods (1-250 days) without reports of ceftriaxone-nonsusceptible infections. We examined scenarios in which more than 1 case was detected initially before detection ceased, from 2 detected cases, as was observed in Massachusetts, up to 10 detected cases.

For each scenario, we calculated 1) the cumulative number of undetected infections with the nonsusceptible strain and 2) the likelihood of elimination of the nonsusceptible strain, defined as the proportion of simulations without new undetected infections.

Analyses were performed using Python 3.9.13. All code is available at https://github.com/gradlab/iceberg.

## Results

### First discovery of the nonsusceptible strain

**Figure 1:**
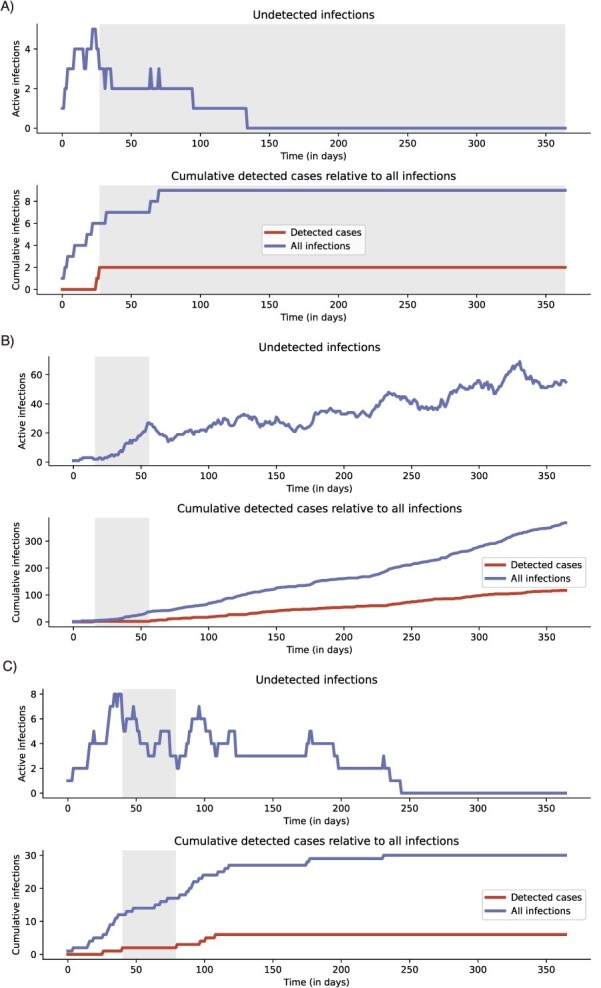
Sample simulation trajectories illustrating the relationship between observed and hidden dynamics. Active undetected infections (top row of each panel) and cumulative detected and total infections (bottom row of each panel) of the nonsusceptible strain over time for 3 sample simulations. Shaded areas (gray) indicate periods in which exactly 2 nonsusceptible cases have been detected. A) Rapid strain elimination. B) Strain persistence. C) Eventual strain elimination.

A diversity of transmission scenarios yielded similar surveillance signals. Three example simulations (Figure 1) showcase the wide range of possible trajectories underlying the detection of 2 cases of the ceftriaxone-nonsusceptible strain, followed by a period without new reports of nonsusceptible infections. The strain may be eliminated shortly after detection of the second case (Figure 1A) or it may persist in the population via continued transmission (Figure 1B). Detection of additional cases was associated either with strain persistence (Figure 1B) or strain elimination (Figure 1C).

**Figure 2:**
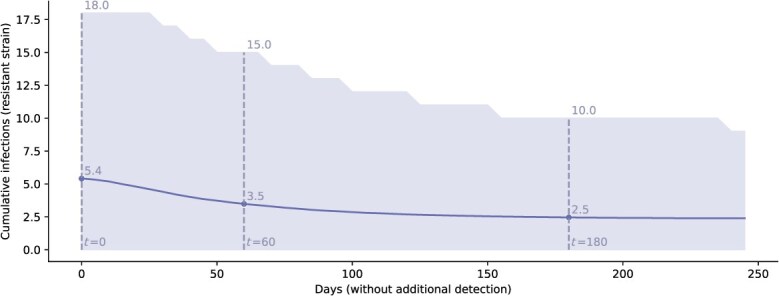
Updating the estimate of the undetected burden of nonsusceptible infections with the passing of time without new reported nonsusceptible infections after detection of 1 case of a ceftriaxone-nonsusceptible strain. Estimate of the cumulative number of undetected nonsusceptible infections as the number of days without observed nonsusceptible cases increases. The solid line represents the average estimate and the shaded area highlights the 95% uncertainty interval. Dashed lines highlight values at 0, 60, and 180 days without detection.

The wide range of possible trajectories was associated with wide uncertainty intervals around initial estimates of the number of unreported infections. Upon detection of the first nonsusceptible case, the total number of undetected nonsusceptible infections was estimated to be 5.4 infections, with a 95% uncertainty interval ranging from 0 to 18 infections (Figure 2). Over time, either additional detections of the new strain or the absence of new detections would narrow the range of possible trajectories and thus increase confidence in estimates of undetected infections. In our simulations, after 6 months (180 days) without any new detections of nonsusceptible infections, the estimate of the cumulative number of unreported infections was reduced to 2.5 with a narrower 95% uncertainty interval of 0-10 infections (Figure 2).

**Figure 3:**
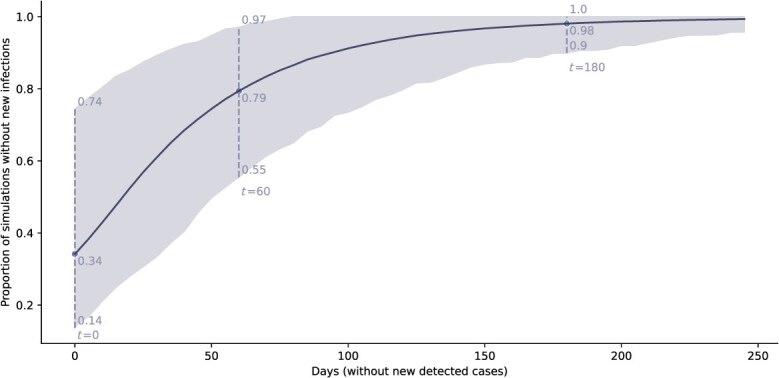
Likelihood of strain elimination for increasing days without detection of ceftriaxone-nonsusceptible cases after detection of 1 case of a ceftriaxone-nonsusceptible strain. The proportion of simulations without new undetected infections for increasing days on which no new cases were detected (mean and 95% uncertainty interval). Dashed lines highlight values at 0, 60, and 180 days without detection.

Increasing time without new detections of nonsusceptible infections also increased the likelihood that the strain had been eliminated. On the day the first nonsusceptible case was detected, the likelihood of elimination was only 34% (95% uncertainty interval, 14-74), meaning the new strain continued causing new infections in 66% of simulations (Figure 3). Each additional day without detection reduced the likelihood that the nonsusceptible strain still in circulation. After 180 days without detection of ceftriaxone-nonsusceptible cases, the strain had been eliminated in 98% of simulations (95% uncertainty interval, 90-100).

### Massachusetts scenario: Detecting 2 nonsusceptible infections

Considering the scenario observed in Massachusetts in 2022, the number of unreported infections upon detection of 2 nonsusceptible cases was estimated to be 10.6 infections (95% uncertainty interval, 2-28) (Figure S2A). In the absence of additional detections of ceftriaxone-nonsusceptible infections over the subsequent 180 days, the estimate declined to 5.7 cumulative undetected infections (95% uncertainty interval, 1-16). The likelihood of strain elimination increased, as no new cases were detected, from 20% (95% uncertainty interval, 7-53) on day 0 to 98% (95% uncertainty interval, 88-100) on day 180 (Figure S2B).

### Comparing scenarios with varying numbers of initially detected cases

**Figure 4:**
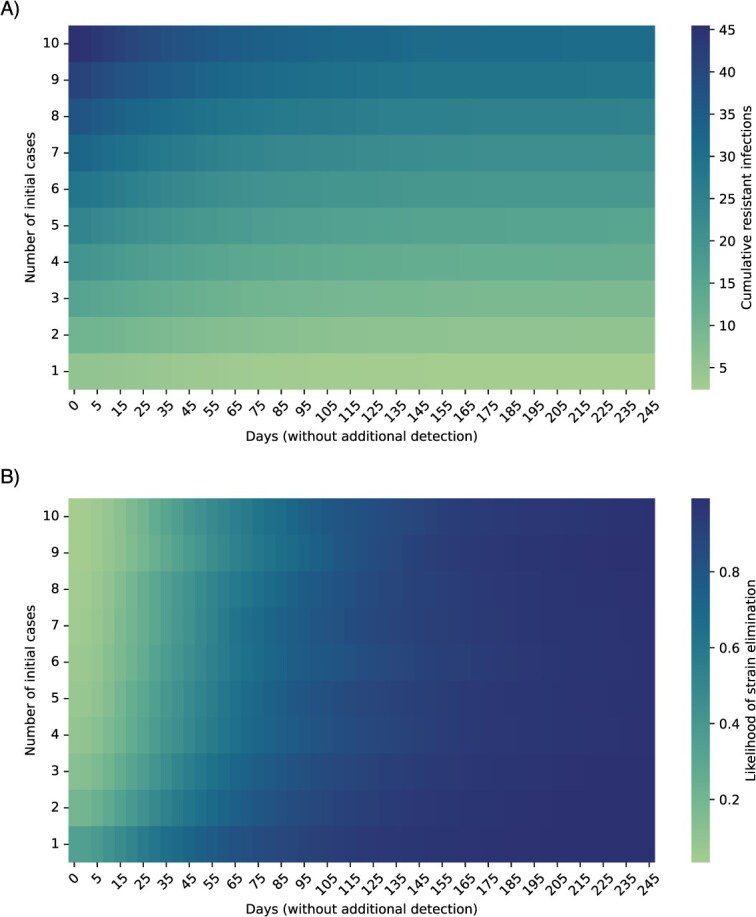
Varying numbers of initial detections followed by increasing periods without new detected cases. Estimates of the average (A) cumulative number of infections and (B) likelihood of strain elimination for scenarios with 1-10 initial detected cases followed by 0-245 days without detection of new cases.

Expanding upon the scenarios with 1 and 2 detections, we examined whether the impact of time without detection depends on the number of cases initially detected. We considered simulations with initial detections ranging from 1 to 10 cases, followed by periods without new reports of nonsusceptible infections, placing no restrictions on the time frame in which these initial infections occurred. The cumulative number of infections varied primarily by the number of initial detections (Figure 4A), whereas the average likelihood of strain elimination varied primarily by the time since the last detected case (Figure 4B). When 1 case was discovered, it took 100 days to be 90% confident that the nonsusceptible strain had been eliminated, whereas when 10 cases had been reported, it took 150 days to achieve 90% elimination likelihood (Figure 4B). Long periods without new detected cases became increasingly unlikely as more initial cases were detected, resulting in wider uncertainty intervals around estimates of the undetected infection burden and the likelihood of strain elimination (Figure S3).

### Comparing strains with varying levels of resistance

**Figure 5:**
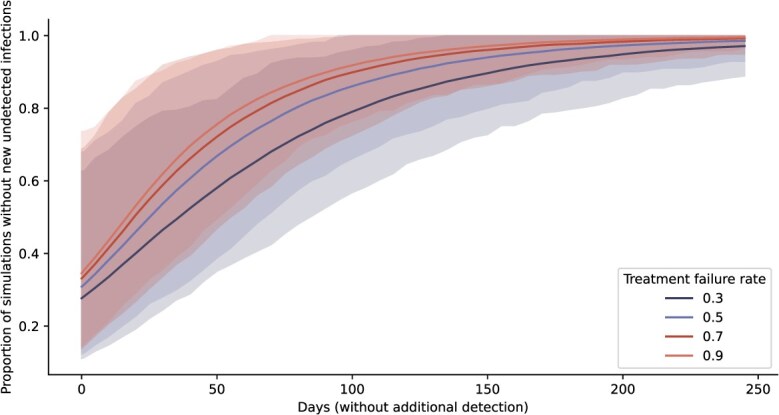
Varying treatment failure rates of a nonsusceptible strain. Likelihood of strain elimination for increasing days without newly reported nonsusceptible cases (mean and 95% uncertainty interval), for treatment failure rates of 30%, 50%, 70%, and 90%.

In our model, nonsusceptible strains could be detected if a specimen was randomly sampled for antibiotic susceptibility testing or through treatment failure. When a strain had a high treatment failure rate, infections were less likely to be successfully treated and, therefore, more likely to be detected, due to either persistent symptoms or test of cure. Higher treatment failure rates, therefore, were associated with fewer unreported infections (Figure S4). Conversely, not detecting any new cases of strains with a high treatment failure rate was more likely a sign that there were, indeed, no new infections, resulting in higher confidence in strain elimination. After 30 days without detecting new cases, a strain with a treatment failure rate of 90% had been eliminated in 62% of simulations (95% uncertainty interval, 36-90), whereas a strain with a treatment failure rate of 30% had only a 47% chance of being eliminated (95% uncertainty interval, 24-79) (Figure 5).

### Comparing varying surveillance intensities

**Figure 6:**
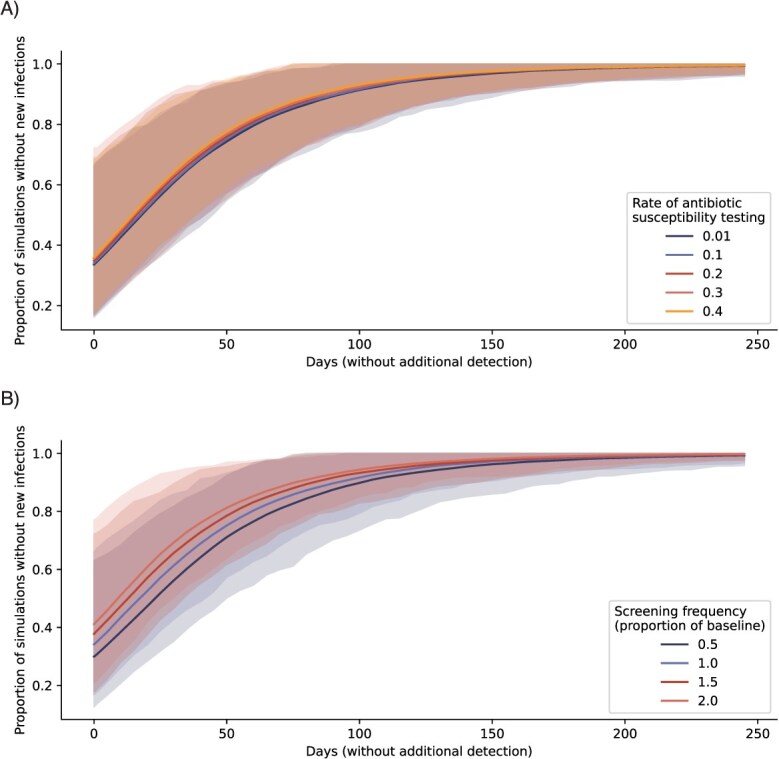
Varying surveillance intensities. Likelihood of strain elimination for increasing days without newly reported nonsusceptible cases (mean and 95% uncertainty interval) for (A) antibiotic susceptibility testing rates ranging from 1% to 40% of all positive gonorrhea diagnoses, and (B) asymptomatic screening rates ranging from 50% to 200% of baseline levels in the calibrated model, preserving the relative screening intensities by population group.

We examined to what extent our estimates of ceftriaxone-nonsusceptible infections depended on antibiotic-susceptibility testing coverage and screening frequency. Increasing levels of surveillance via increased antibiotic susceptibility testing and asymptomatic screening both reduced the estimated number of undetected infections, though the effect of increasing screening was stronger (Figure S5). Doubling the baseline screening intensity (with fixed antibiotic susceptibility testing of 10%) lowered the estimated undetected burden on the day of the first detection from 5.1 (95% uncertainty interval, 0-17) to 4.3 infections (95% uncertainty interval, 0-15), whereas doubling the rate of antibiotic susceptibility testing lowered the estimated undetected infections to only 4.8 (95% uncertainty interval, 0-16) (Figure S5). Increased surveillance also reduced the likelihood of ongoing transmission in the absence of detection, again with a stronger effect of asymptomatic screening over antibiotic susceptibility testing. Doubling screening frequency increased the likelihood of strain elimination from 34% (95% uncertainty interval, 17-66) to 41% (21-77), whereas doubling the antibiotic susceptibility testing rate raised the likelihood to 35% (95% uncertainty interval, 17-69) (Figure 6).

## Discussion

To help guide the surveillance of AMR, we estimated how many infections evade detection when a new ceftriaxone-nonsusceptible strain of *N. gonorrhoeae* is first detected, taking the reports of 2 multidrug nonsusceptible infections in Massachusetts as a case study. In our simulations, a wide range of disease scenarios generated similar surveillance signals that were consistent with 2 detected cases followed by a period without detection; these hidden dynamics ranged from rapid strain elimination to persistence of the strain in the population. This diversity of outbreak trajectories compatible with the same surveillance signal illustrates how difficult it can be to interpret both days with and without detection and to anticipate whether newly detected strains of concern will continue to spread in a given location.

We estimated that on the day a ceftriaxone-nonsusceptible strain was first discovered in Massachusetts, the undetected burden was 5.4 infections and in the range of 0 to 18 cases. By the time 2 infections had been detected, an estimated 10.6 infections had evaded detection, with a likely range of 2-28 infections. Transmission was ongoing in 66% of simulations the day the first case was detected and declined to 2% after 180 days (6 months) without new reports of nonsusceptible cases. In simulations with 2 detected infections, 80% of simulations had ongoing transmission the day the second case was detected, and the likelihood of strain persistence also declined to 2% after 180 days without new reports of ceftriaxone-nonsusceptible infections.

The link between surveillance signals and the true disease dynamics was stronger for highly resistant strains. When strains had a higher risk of treatment failure, the absence of detection was more indicative of an underlying absence of transmission than for intermediately resistant strains. This finding suggests the phenotypic characteristics of the novel strain should be considered in the interpretation of surveillance data.

As more nonsusceptible cases were found, the gain in elimination probability from each day without new cases decreased. When only 1 case had been detected, it took 100 days to be 90% confident that the strain had been eliminated. But if 10 cases had initially been reported, the likelihood of strain elimination after 100 days without detection was only 76%, and it would take 150 days without detection to reach 90% confidence. This finding emphasizes the importance of responding quickly to the first detected cases of a new strain of concern.

Increasing the asymptomatic screening frequency reduced the undetected infection burden and thus increased surveillance accuracy. Greater screening coverage also reduced the likelihood of ongoing transmission in the absence of detection, likely due to the longer infectious period of asymptomatic infections, which can produce new transmission events even after a period without new detections of nonsusceptible infections These results are relevant for decision-making about asymptomatic screening recommendations, because they illustrate the complex impacts of screening on antibiotic use and surveillance of resistance.

Our findings rely on several modeling assumptions. We maintained many of the structural elements in prior gonorrhea models^24^^-^^27^ and added complexity only as necessary. Many models of gonorrhea focus on a single subpopulation, such as MSM.^24^^,^^28^ We modeled both heterosexual and homosexual contact networks jointly, because ceftriaxone resistance has been observed in heterosexual men and women,^8^ and gonorrhea prevalence is highest among MSM.^29^ However, we did not explicitly account for other population heterogeneities such as age structure, geography, or infection prevalence by anatomic site, because we did not have sufficiently confident estimates of parameters required to construct such a model.

The rate at which gonorrhea infections are imported to Massachusetts (and globally) is poorly understood. In this model, we measured outcomes for a single importation event, a realistic assumption if importations of nonsusceptible strains are sufficiently infrequent. Estimates of the cumulative burden and likelihood of strain elimination will differ if multiple independent importations occur or if nonsusceptible infections are replenished before the strain is eliminated. Given that reduced susceptibility to ceftriaxone has been reported only 3 times in the United States to date,^13^^,^^14^ it is probable that the 2 infections observed in Massachusetts were linked. We assumed that importation may occur in any of our 8 subpopulations, because the relative contribution of different genders, genders of sex partners, and sexual activity groups to strain importation has not yet been characterized. Once more data become available, the simulations should be reweighted to account for differences in importation frequency. We also note that although we focused here on importations of ceftriaxone-nonsusceptible strains, our results would also apply to de novo mutations originating within the population.^5^

With increasing global reports of nonsusceptibility to ceftriaxone, the last remaining antibiotic for empiric treatment of gonorrhea, challenges in treating gonococcal infections are a serious public health threat. Scaling up surveillance of AMR in gonorrhea is costly, given the challenges with culture-based surveillance, asymptomatic infections, and contact tracing. To optimize the use of limited resources, we need tools to help estimate the infection landscapes that could underlie the data from our surveillance systems. The inferential approach reported here helps fill this need, both in interpreting the data from Massachusetts and in establishing a platform for other settings.

### Supplementary material

Supplementary material is available at the *American Journal of Epidemiology* online.

## Supplementary Material

Web_Material_kwae455

## Data Availability

All code is available at https://github.com/gradlab/iceberg.
