# Supplementary material for: Estimating the undetected burden and the likelihood of strain persistence of drug-resistant Neisseria gonorrhoeae
Source: Am J Epidemiol. 2024 Dec 13;194(10):2861–9. doi: 10.1093/aje/kwae455 (PMC12527230; doi:10.1093/aje/kwae455)
Supplement: Web_Material_kwae455 [file web_material_kwae455.zip › Supplemental Materials and Methods_2024-12-03.docx]

**Supplementary Material**

**Estimating the undetected burden and the likelihood of strain persistence of drug-resistant Neisseria gonorrhoeae**

Kirstin I. Oliveira Roster, Minttu M. Rönn, Heather Elder, Thomas L. Gift, Kathleen A. Roosevelt, Joshua A. Salomon, Katherine K. Hsu, Yonatan H. Grad

Table of Contents

Figure S1 1

Appendix S1 2

Table S1 5

Table S2 6

Table S3 8

Figure S2 9

Figure S3 10

Figure S4 10

Figure S5 11

**Figure S1. Compartmental model structure**

**Appendix S1**

Compartmental model equations

Susceptible

$\frac{dS}{dt}$ = + population entry - population exit – infection + innate recovery + recovery upon treatment + retreatment

$$\frac{dS}{dt}= \mu_{entry}N -\mu_{exit}S-\frac{\boldsymbol{\beta I}S}{N}+\frac{1}{D_{n}}\left( \boldsymbol{I}_{\boldsymbol{inc}}+\boldsymbol{I}_{\boldsymbol{a}}+\boldsymbol{I}_{\boldsymbol{s}}\boldsymbol{+ I}_{\boldsymbol{treat, fail}} \right)+\frac{1}{D_{r}}\boldsymbol{I}_{\boldsymbol{treat,success}}+p_{screen} I_{a,r, treat,fail}$$

Symptomatic infection with resistant strain:

$\frac{dI_{inc,r}}{dt}$ = - population exit + infection – innate recovery – symptom onset

$$\frac{dI_{inc,r}}{dt}=p_{symptoms}\frac{\boldsymbol{\beta}S\boldsymbol{I}_{\boldsymbol{r}}}{N}-\frac{1}{D_{n}}I_{inc,r}-\frac{1}{D_{inc}}I_{inc,r}-\mu_{exit}I_{inc,r}$$

$\frac{dI_{s,r}}{dt}$ = symptom onset – innate recovery – treatment – population exit

$$\frac{dI_{s,r}}{dt}=\frac{1}{D_{inc}}I_{inc,r}-\frac{1}{D_{n}}I_{s,r}-\frac{1}{D_{treat}} I_{s,r}-\mu_{exit}I_{s,r}$$

$\frac{dI_{s,r,treat,success}}{dt}$ = successful treatment – recovery upon treatment – population exit

$$\frac{dI_{s,r,treat,success}}{dt}=\left( 1-p_{tf} \right)\frac{1}{D_{treat}} (1-p_{d})I_{s,r}-\frac{1}{D_{r}}I_{s,r,treat,success}- \mu_{exit}I_{s,r,treat,success}$$

$\frac{dI_{s,r,treat,fail}}{dt}$ = failed treatment – retreatment – innate recovery - population exit

$$\frac{dI_{s,r,treat,fail}}{dt}=\frac{p_{tf}}{D_{treat}}(1-p_{d})I_{s,r}-\frac{1}{D_{rt}}I_{s,r,treat,fail}-\frac{1}{D_{n}}I_{s,r,treat,fail}- \mu_{exit}I_{s,r,treat,fail}$$

$\frac{dI_{s,r,detect}}{dt}$= detection at first treatment + retreatment – recovery upon treatment – population exit

$$\frac{dI_{s,r,detect}}{dt}=\frac{1}{D_{treat}}p_{d}I_{s,r}+ \frac{1}{D_{rt}}I_{s,r,treat,fail}-\frac{1}{D_{r}}I_{s,r,detect}- \mu_{exit}I_{s,r,detect}$$

Asymptomatic infection with resistant strain:

$\frac{dI_{a,r}}{dt}$= infection – innate recovery – treatment – population exit

$$\frac{dI_{a,r}}{dt}={(1-p}_{symptoms}) \frac{\boldsymbol{\beta}\boldsymbol{I}_{\boldsymbol{r}}S}{N}-\frac{1}{D_{n}}I_{a,r}-\frac{1}{D_{screen}} I_{a,r}-\mu_{exit} I_{a,r}$$

$\frac{dI_{a,r,treat,success}}{dt}$= successful treatment – recovery upon treatment – population exit

$$\frac{dI_{a,r,treat,success}}{dt}=\left( 1-p_{tf} \right) \left( 1-p_{d} \right) \frac{1}{D_{screen}} I_{a,r}-\frac{1}{D_{r}}I_{a,r,treat,success}- \mu_{exit}I_{a,r,treat,success}$$

$\frac{dI_{a,r,treat,fail}}{dt}$= failed treatment – innate recovery - retreatment – population exit

$$\frac{dI_{a,r,treat,fail}}{dt}=p_{tf}\left( 1-p_{d} \right) \frac{1}{D_{screen}} I_{a,r}-\frac{1}{D_{n}} I_{a,r,treat,fail}- {(p}_{TOC}+\frac{1}{D_{screen}}) I_{a,r,treat,fail}-\mu_{exit} I_{a,r,treat,fail}$$

$\frac{dI_{a,r,detect}}{dt}$= detection at first treatment + retreatment – recovery upon treatment – population exit

$$\frac{dI_{a,r,detect}}{dt}=p_{d}\frac{1}{D_{screen}} I_{a,r}+p_{TOC} I_{a,r,treat,fail}-\frac{1}{D_{r}}I_{a,r,detect}- \mu_{exit}I_{a,r,detect}$$

Symptomatic infection with non-resistant strain:

$\frac{dI_{inc,nr}}{dt}$ = - population exit + infection – innate recovery – symptom onset

$$\frac{dI_{inc,nr}}{dt}=p_{symptoms}\frac{\boldsymbol{\beta}S\boldsymbol{I}_{\boldsymbol{nr}}}{N}-\frac{1}{D_{n}}I_{inc,nr}-\frac{1}{D_{inc}}I_{inc,nr}-\mu_{exit}I_{inc,nr}$$

$\frac{dI_{s,nr}}{dt}$ = symptom onset – innate recovery – treatment – population exit

$$\frac{dI_{s,nr}}{dt}=\frac{1}{D_{inc}}I_{inc,nr}-\frac{1}{D_{n}}I_{s,nr}-\frac{1}{D_{treat}} I_{s,nr}-\mu_{exit}I_{s,nr}$$

Asymptomatic infection with non-resistant strain:

$\frac{dI_{a,nr}}{dt}$= infection – innate recovery – treatment – population exit

$$\frac{dI_{a,nr}}{dt}={(1-p}_{symptoms}) \frac{\boldsymbol{\beta}\boldsymbol{I}_{\boldsymbol{nr}}S}{N}-\frac{1}{D_{n}}I_{a,nr}-\frac{1}{D_{screen}} I_{a,nr}-\mu_{exit} I_{a,nr}$$

$\frac{dI_{nr,treat,success}}{dt}$ = treatment – recovery upon treatment – population exit

$$\frac{dI_{nr,treat,success}}{dt}=\left( 1-p_{d} \right)\frac{1}{D_{treat}} I_{s,nr}+\left( 1-p_{d} \right) \frac{1}{D_{screen}} I_{a,nr}-\frac{1}{D_{r}}I_{nr,treat,success}- \mu_{exit}I_{nr,treat,success}$$

$\frac{dI_{nr,detect}}{dt}$ = detection at first treatment – recovery upon treatment – population exit

$$\frac{dI_{nr,detect}}{dt}=p_{d} \frac{1}{D_{treat}} I_{s,nr}+p_{d} \frac{1}{D_{screen}} I_{a,nr}-\frac{1}{D_{r}}I_{nr,detect}- \mu_{exit}I_{nr,detect}$$

Where:

Mixing matrix

$$\beta_{ij}=b_{ij}m_{i\to j}^{*}$$

$m_{i\to j}=\epsilon c_{i}N_{i}\frac{c_{j}N_{j}}{\sum_{k\in K_{a}} c_{k}N_{k}}$ for $i,j$in same activity group, and $m_{i\to j}=(1-\epsilon) c_{i}N_{i}\frac{c_{j}N_{j}}{\sum_{k\in K_{d}} c_{k}N_{k}}$ for $i,j$in different activity groups, where $c_{i}$ is the average number of partners for individuals in group $i$, $N_{i}$ is the population size of group $i$, $\epsilon$ is the assortative mixing parameter, and $b_{ij}$ is the per-partnership transmission probability given an infectious contact. Mixing is defined in this way for activity groups within MSM, between MSW and WSM, between MSMW and MSM, and between MSMW and WSM.

Mixing is adjusted to balance supply and demand for partnerships:

$m_{i\to j}^{*}=B^{\theta-1}m_{i\to j}$

$m_{j\to i}^{*}=B^{\theta}m_{j\to i}$

where $\theta$is a balancing parameter and $B=\frac{m_{i\to j}}{m_{j\to i}}$.

Notation

$$\boldsymbol{I}=\boldsymbol{I}_{\boldsymbol{inc}}\boldsymbol{+}\boldsymbol{I}_{\boldsymbol{s}}\boldsymbol{+}\boldsymbol{I}_{\boldsymbol{a}}\boldsymbol{+}\boldsymbol{I}_{\boldsymbol{treat, success}}\boldsymbol{+}\boldsymbol{I}_{\boldsymbol{treat,fail}}$$

$$\boldsymbol{I}_{\boldsymbol{inc}}=I_{inc,r}+I_{inc, nr}$$

$$\boldsymbol{I}_{\boldsymbol{a}}=I_{a,r}+I_{a,nr}$$

$$\boldsymbol{I}_{\boldsymbol{s}}=I_{s,r}+I_{s,nr}$$

$$\boldsymbol{I}_{\boldsymbol{treat, fail}}=I_{a,r,treat, fail}+I_{s,r,treat,fail}$$

$$\boldsymbol{I}_{\boldsymbol{treat,success}}=I_{a,r,treat, success}+I_{a,nr,treat,success}+I_{s,r,treat,success}+I_{s,nr,treat,success}$$

Compartments:

$S$: Susceptible

$I$: Infectious

Subscripts:

$r$: resistant strain

$nr$: non-resistant strain

$a$: asymptomatic

$inc$: incubation period

$s$: symptomatic

$treat, success$: successful treatment

$treat, fail$: failed treatment

$detect$: treatment of infection and antibiotic susceptibility testing of strain

**Table S1. Fitted Parameters**

| **Parameter** | **Description** | **Group** | **Prior distribution** | **Prior mean** | **Reference** | **Posterior mean (75% CI)** |
| --- | --- | --- | --- | --- | --- | --- |
| $p_{symptoms}$ | Proportion of infections that are symptomatic | Men | Beta(4.3, 2.3) | 0.65 | MDPH | 0.40 (0.31-0.51) |
|  |  | Women | Beta(6.2, 5.3) | 0.54 | MDPH | 0.53 (0.45-0.64) |
| $c_{i,high}$ for $i\in\{MSM,$  $MSMW, MSW,$  $WSM\}$ | Average number of contacts (per year), high activity groups | MSM | Gamma(9.3, 3.3) | 30.5 | (12), DPH | 30.9 (23.3-38.7) |
|  |  | MSMW | Male: Gamma(5.2,4.4)  Female: Gamma(3.8, 4.0) | Male: 30.5*0.75  Female: 20*0.75 | Assumption | Male: 28.6 (17.6-37.2)  Female: 18.7 (11.6-22.7) |
|  |  | MSW | Gamma(6.7, 3.0) | 20 | (5,13), MDPH | 18.4 (11.8-21.9) |
|  |  | WSM | Gamma(6.7, 3.0) | 20 | (5,13), MDPH | 20.5 (16.8-25.7) |
| $p_{L,i}$ where  ${{c_{i,low}= p}_{L,i}c}_{i,high}$ for $i\in\{MSM,$  $MSMW, MSW,$  $WSM\}$ | Average number of contacts (per) year in low activity group, as a proportion of high activity group | MSM | Uniform(0,1) | 0.5 |  | 0.48 (0.25-0.68) |
|  |  | MSMW | Uniform(0,1) | 0.5 |  | Male: 0.58 (0.50-0.73)  Female: 0.48 (0.27-0.72) |
|  |  | MSW | Uniform(0,1) | 0.5 |  | 0.41 (0.14-0.66) |
|  |  | WSM | Uniform(0,1) | 0.5 |  | 0.40 (0.10-0.64) |
| $\epsilon$ | Assortative mixing parameter |  | Beta(2,2) | 0.5 | (14) | 0.49 (0.30-0.69) |
| $b_{ij}$ | Per-partnership transmission probability given infectious contact between groups $i,j$ | MSM, low-low | Beta(13.7, 9.5) | 0.59 | (12,15) | 0.61 (0.55-0.69) |
|  |  | MSM, high-high | Beta(12.0,12.0) | 0.5 | (3,12,15) | 0.53 (0.47-0.59) |
|  |  | Male-to-female, low-low | Beta(12.0,3.0) | 0.8 | (12,13) | 0.80 (0.74-0.89) |
|  |  | Male-to-female, high-high | Beta(13.8, 5.4) | 0.72 | (12) | 0.72 (0.64-0.79) |
|  |  | Female-to-male, low-low | Beta(12.0,3.0) | 0.8 | (12,13) | 0.77 (0.73-0.82) |
|  |  | Female-to-male, high-high | Beta(13.8, 5.4) | 0.72 | (12) | 0.73 (0.68-0.80) |
| $D_{treat}$ | Duration from start of symptoms to treatment, in days | Men | Gamma(6.7, 0.8) | 5.3 | MDPH | 5.6 (4.1-6.4) |
|  |  | Women | Gamma(13.6, 0.6) | 8.4 | MDPH | 8.5 (7.1-10.0) |
| $D_{r}$ | Duration from time of treatment to recovery | Men | Gamma(3.0, 1.0) | 3 | (16) | 3.2 (2.2-4.0) |
|  |  | Women | Gamma(3.0, 1.0) | 3 | (16) | 3.0 (1.8-3.6) |
| $D_{n}$ | Duration from infection to natural clearance | Men | Gamma(484.0, 0.1) | 44 | (17–19) | 44.1 (42.7-45.3) |
|  |  | Women | Gamma(1936.0, 0.05) | 88 | (17–19) | 87.7 (86.5-87.7) |
| $D_{inc}$ | Duration of incubation period | Men | Gamma(22.4, 0.3) | 6.7 | (17,18,20) | 6.8 (6.1-7.6) |
|  |  | Women | Gamma(36.0, 0.3) | 12 | (17,18,21) | 12.2 (10.8-13.0) |
| $F_{screen,i}$, where $D_{screen,high,i}=\frac{365}{F_{screen,i}}$ for $i\in\{MSM,$  $MSMW, MSW,$  $WSM\}$ | Average asymptomatic screening frequency (per year) | MSM, high activity | Uniform(1,4) | 2.5 | (22) | 2.5 (1.7 - 3.2) |
|  |  | MSMW, high activity | Uniform(1,4) | 2.5 | (22) | 2.3 (1.8 – 2.8) |
|  |  | MSW, high activity | Uniform(0,1) | 0.5 | (22) | 0.57 (0.37-0.86) |
|  |  | WSM, high activity | Uniform(0,1) | 0.5 | (22) | 0.44 (0.23-0.65) |
| $p_{screen,low,i}$ where $D_{screen,low,i}=\frac{365}{{p_{screen,low,i} F}_{screen,i}}$ for $i\in\{MSM,$  $MSMW, MSW,$  $WSM\}$ | Average asymptomatic screening frequency, (per year) | MSM, low activity | Uniform(0,1) * $F_{screen,MSM}$ | 0.5 * $F_{screen,MSM}$ |  | 1.4 (0.6-2.0) |
|  |  | MSMW, low activity | Uniform(0,1) * $F_{screen,MSMW}$ | 0.5 * $F_{screen,MSMW}$ |  | 1.1 (0.6-1.7) |
|  |  | MSW, low activity | Uniform(0,1) * $F_{screen,MSW}$ | 0.5 * $F_{screen,MSW}$ |  | 0.3 (0.1-0.4) |
|  |  | WSM, low activity | Uniform(0,1) * $F_{screen,WSM}$ | 0.5 * $F_{screen,WSM}$ |  | 0.2 (0.1-0.3) |
| $p_{detect}$ | Antibiotic susceptibility testing probability (per positive sample) | Men | Uniform(0,0.3) | 0.15 | Assumption, MDPH | 0.05 (0.03-0.06) |
|  |  | Women | Uniform(0,0.3) | 0.15 | Assumption, MDPH | 0.03 (0.01-0.04) |

Abbreviations: Men who have sex with men (MSM), men who have sex with men and women (MSMW), men who have sex with women (MSW), women who have sex with men (WSM).

**Table S2. Fixed Parameters**

| **Parameter** | **Description** | **Group** | **Value** | **Source** |
| --- | --- | --- | --- | --- |
| $N$ | Population size |  | 5,679,309 | MDPH |
|  | Population proportion, by group | MSM, high activity | 0.025 * 0.15 | Assumptions, following (1–4) |
|  |  | MSM, low activity | 0.025 * 0.85 |  |
|  |  | MSMW, high activity | 0.025 * 0.15 |  |
|  |  | MSMW, low activity | 0.025 * 0.85 |  |
|  |  | MSW, high activity | 0.475 * 0.15 |  |
|  |  | MSW, low activity | 0.475 * 0.85 |  |
|  |  | WSM, high activity | 0.475 * 0.15 |  |
|  |  | WSM, low activity | 0.475 * 0.85 |  |
| $\mu_{entry}, \mu_{exit}$ | Rates of population entry and exit, per person per day |  | 1/(20*365) | (3,5) |
|  | Initial gonorrhea prevalence | MSM (all) | 0.03 | Assumption, following (3) |
|  |  | MSM, high activity | 0.08 | Assumption |
|  |  | Men | 0.0126 | (6) |
|  |  | Women | 0.0161 | (6) |
|  |  | Women, high activity | 0.027 | (7,8) |
| $p_{TOC}$ | Rate of follow-up for test of cure after asymptomatic screening | Men | 0.22 / 10 | (9–11) |
|  |  | Women | 0.38 / 10 | (9) |
| $D_{rt}$ | Time until retreatment, in days, given treatment failure of symptomatic infection | Men | 7 | Assumption |
|  |  | Women | 7 | Assumption |
| $p_{tf}$ | Probability of treatment failure if resistant strain is treated with ceftriaxone |  | 0.8 | Assumption |

Abbreviations: Men who have sex with men (MSM), men who have sex with men and women (MSMW), men who have sex with women (MSW), women who have sex with men (WSM).

**Table S3. Calibration targets**

| **Target** | **Group** | **Accepted range** | **Source** |
| --- | --- | --- | --- |
| Annual observed incidence, per 100,000 population | Men | $(0.75*181;1.25*181 )$ | MDPH |
|  | Women | $(0.75*80;1.25*80)$ | MDPH |
| Prevalence, per population | MSM | $(0.02;0.06)$ | (3) |
|  | MSW | $(0.003;0.02)$ | (12,23–25) |
|  | WSM | $(0.003;0.02)$ | (12,23–25) |

Abbreviations: Men who have sex with men (MSM), men who have sex with men and women (MSMW), men who have sex with women (MSW), women who have sex with men (WSM).

**Figure S2: Disease burden and likelihood of strain elimination for increasing days without detection of resistant cases, following the detection of 2 resistant cases**

(A) Estimate of the number of undetected infections with the resistant strain after increasing days without newly reported resistant cases (mean and 95% uncertainty interval).

(B) Proportion of simulations without new cases for increasing days without newly reported resistant cases (mean and 95% uncertainty interval). Dashed lines highlight values at 0, 60, and 180 days without detection.

**A)**

**B)**

**Figure S3. Confidence in elimination for varying levels of detected cases.** Proportion of simulations without new cases for increasing days without newly reported resistant cases after initially detecting 1, 5, and 10 resistant cases (mean and 95% uncertainty interval).

**Figure S4. Undetected disease burden for varying treatment failure rates.** Undetected non-susceptible infections upon detection of one non-susceptible case and increasing days without newly detected cases (mean and 95% uncertainty interval), for varying treatment failure rates.

**Figure S5. Undetected disease burden for varying surveillance intensities.** Undetected non-susceptible infections upon detection of one non-susceptible case and increasing days without newly detected cases (mean and 95% uncertainty interval), for (A) varying antibiotic susceptibility testing rates and (B) varying asymptomatic screening intensities.

1. **Varying antibiotic susceptibility testing rates**

**B) Varying asymptomatic screening intensities**

**References**

1. Mather D, Murphy J, Mehta A, Manukyan M, Dooley D. Healthcare Access, Health Behaviors, and Health Status Among Lesbian, Gay, Bisexual, and Transgender Adults in Boston, 2010 – 2017. Boston Public Health Commission; 2019.

2. Grey JA, Bernstein KT, Sullivan PS, Purcell DW, Chesson HW, Gift TL, et al. Estimating the Population Sizes of Men Who Have Sex With Men in US States and Counties Using Data From the American Community Survey. JMIR Public Health Surveill. 2016 Apr 21;2(1):e5365.

3. Reichert E, Yaesoubi R, Rönn MM, Gift TL, Salomon JA, Grad YH. Resistance-minimising strategies for introducing a novel antibiotic for gonorrhoea treatment: a mathematical modelling study. Lancet Microbe. 2023 Oct 1;4(10):e781–9.

4. Farmer GW, Bucholz KK, Flick LH, Burroughs TE, Bowen DJ. CVD Risk among Men Participating in the National Health and Nutrition Examination Survey (NHANES) from 2001–10: Differences by Sexual Minority Status. J Epidemiol Community Health. 2013 Sep;67(9):10.1136/jech-2013–202658.

5. Hazel A, Marino S, Simon C. An anthropologically based model of the impact of asymptomatic cases on the spread of Neisseria gonorrhoeae. J R Soc Interface. 2015 May 6;12(106):20150067.

6. Pollock ED, Clay PA, Kreisel KM, Spicknall IH. Estimated Incidence and Prevalence of Gonorrhea in the United States, 2006-2019. Sex Transm Dis. 2023 Apr 1;50(4):188–95.

7. Whelan J, Abbing-Karahagopian V, Serino L, Unemo M. Gonorrhoea: a systematic review of prevalence reporting globally. BMC Infect Dis. 2021 Nov 11;21(1):1152.

8. Mc Grath-Lone L, Marsh K, Hughes G, Ward H. The sexual health of female sex workers compared with other women in England: analysis of cross-sectional data from genitourinary medicine clinics. Sex Transm Infect. 2014 Jun;90(4):344–50.

9. Hoover KW, Tao G, Nye MB, Body BA. Suboptimal Adherence to Repeat Testing Recommendations for Men and Women With Positive Chlamydia Tests in the United States, 2008–2010. Clin Infect Dis. 2013 Jan 1;56(1):51–7.

10. Rose SB, Garrett SM, Stanley J, Pullon SRH. Retesting and repeat positivity following diagnosis of Chlamydia trachomatis and Neisseria gonorrhoea in New Zealand: a retrospective cohort study. BMC Infect Dis. 2017 Jul 28;17(1):526.

11. Teixeira da Silva D, Petsis D, Santos T, Mahajan A, Bonett S, Wood S. Chlamydia Trachomatis/Neisseria Gonorrhea Retesting Among Adolescents and Young Adults in a Primary Care Network. J Adolesc Health. 2022 Nov 1;71(5):545–51.

12. Fingerhuth SM, Bonhoeffer S, Low N, Althaus CL. Antibiotic-Resistant Neisseria gonorrhoeae Spread Faster with More Treatment, Not More Sexual Partners. PLOS Pathog. 2016 May 19;12(5):e1005611.

13. Garnett GP, Mertz KJ, Finelli L, Levine WC, St Louis ME. The transmission dynamics of gonorrhoea: modelling the reported behaviour of infected patients from Newark, New Jersey. Philos Trans R Soc B Biol Sci. 1999 Apr 29;354(1384):787–97.

14. Zhang L, Regan DG, Chow EPF, Gambhir M, Cornelisse V, Grulich A, et al. Neisseria gonorrhoeae Transmission Among Men Who Have Sex With Men: An Anatomical Site-Specific Mathematical Model Evaluating the Potential Preventive Impact of Mouthwash. Sex Transm Dis. 2017 Oct;44(10):586–92.

15. Kirkcaldy RD, Weston E, Segurado AC, Hughes G. Epidemiology of gonorrhoea: a global perspective. Sex Health. 2019 Sep;16(5):401–11.

16. Barbee LA, Soge OO, Khosropour CM, LeClair A, Golden MR. Time to Clearance of Neisseria gonorrhoeae RNA at the Pharynx following Treatment. J Clin Microbiol. 2022 Jun 15;60(6):e0039922.

17. Li Y, You S, Lee K, Yaesoubi R, Hsu K, Gift TL, et al. The Estimated Lifetime Quality-Adjusted Life-Years Lost Due to Chlamydia, Gonorrhea, and Trichomoniasis in the United States in 2018. J Infect Dis. 2023 Apr 18;227(8):1007–18.

18. Kreisel KM, Weston EJ, St Cyr SB, Spicknall IH. Estimates of the Prevalence and Incidence of Chlamydia and Gonorrhea Among US Men and Women, 2018. Sex Transm Dis. 2021 Apr 1;48(4):222–31.

19. Stupiansky NW, Van Der Pol B, Williams JA, Weaver B, Taylor SE, Fortenberry JD. The natural history of incident gonococcal infection in adolescent women. Sex Transm Dis. 2011 Aug;38(8):750–4.

20. Lovett A, Duncan JA. Human Immune Responses and the Natural History of Neisseria gonorrhoeae Infection. Front Immunol. 2018;9:3187.

21. Korenromp EL, Sudaryo MK, de Vlas SJ, Gray RH, Sewankambo NK, Serwadda D, et al. What proportion of episodes of gonorrhoea and chlamydia becomes symptomatic? Int J STD AIDS. 2002 Feb;13(2):91–101.

22. Centers for Disease Control and Prevention. Sexually Transmitted Infections Treatment Guidelines, 2021 [Internet]. 2021 Jun [cited 2024 May 3]. Available from: https://www.cdc.gov/std/treatment-guidelines/toc.htm

23. Rowley J, Vander Hoorn S, Korenromp E, Low N, Unemo M, Abu-Raddad LJ, et al. Chlamydia, gonorrhoea, trichomoniasis and syphilis: global prevalence and incidence estimates, 2016. Bull World Health Organ. 2019 Aug 1;97(8):548-562P.

24. Torrone EA, Johnson RE, Tian LH, Papp JR, Datta SD, Weinstock HS. Prevalence of Neisseria gonorrhoeae among persons 14 to 39 years of age, United States, 1999 to 2008. Sex Transm Dis. 2013 Mar;40(3):202–5.

25. Satterwhite CL, Torrone E, Meites E, Dunne EF, Mahajan R, Ocfemia MCB, et al. Sexually Transmitted Infections Among US Women and Men: Prevalence and Incidence Estimates, 2008. Sex Transm Dis. 2013 Mar;40(3):187.
